# Central Nervous System Associated With Light Perception and Physiological Responses of Birds

**DOI:** 10.3389/fphys.2021.723454

**Published:** 2021-10-21

**Authors:** Seong W. Kang

**Affiliations:** Department of Poultry Science, Center of Excellence for Poultry Science, University of Arkansas, Fayetteville, AR, United States

**Keywords:** light, melanopsin (Opn4), premammillary nucleus, ventral tegmental area, raphe nucleus, dopamine, serotonin, welfare

## Abstract

Environmental light that animal receives (i.e., photoperiod and light intensity) has recently been shown that it affects avian central nervous system for the physiological responses to the environment by up or downregulation of dopamine and serotonin activities, and this, in turn, affects the reproductive function and stress-related behavior of birds. In this study, the author speculated on the intriguing possibility that one of the proposed avian deep-brain photoreceptors (DBPs), i.e., melanopsin (Opn4), may play roles in the dual sensory-neurosecretory cells in the hypothalamus, midbrain, and brain stem for the behavior and physiological responses of birds by light. Specifically, the author has shown that the direct light perception of premammillary nucleus dopamine-melatonin (PMM DA-Mel) neurons is associated with the reproductive activation in birds. Although further research is required to establish the functional role of Opn4 in the ventral tegmental area (VTA), dorsal raphe nucleus, and caudal raphe nucleus in the light perception and physiological responses of birds, it is an exciting prospect because the previous results in birds support this hypothesis that Opn4 in the midbrain DA and serotonin neurons may play significant roles on the light-induced welfare of birds.

## Introduction

Light perception and integration of photic information in the diurnal animals are critical for their proper adaptation to the environment, and therefore, animals can respond to daily and annual environmental change (Chmura et al., 2019; Hussein et al., 2021). Light plays a central role in modulating animal behavior and is a critical environmental factor that can affect the physiological processes, performance, and welfare of many animals and birds (Wilson and Cunningham, 1980; Manser, 1996; Deep et al., 2010; Fernandes et al., 2013; Aulsebrook et al., 2021). The physiological roles and effects of light include facilitating sight, regulating reproductive hormone release, and affecting social behavior. The most visible physiological effects of light on birds are the effect of photoperiod and light intensity on the seasonal reproduction, health, and behavior of birds (Deep et al., 2010; Olanrewaju et al., 2018; ViviD and Bentley, 2018).

Several studies provide evidence that light can affect the central physiology of animals independent of retinal function (Chiu et al., 1975; Routtenberg et al., 1978; Underwood et al., 1984; Wade et al., 1988; Fernandes et al., 2013). In avian species, photoperiodic synchronization is achieved independently of the pineal melatonin through direct light perception by avian deep-brain photoreceptors (DBPs), which project directly to the median eminence near the pars tuberalis (PT) in the anterior pituitary (Kang et al., 2010; Nakane et al., 2010; Chmura et al., 2019). However, evidence is not available regarding the pathway used by the photoperiodic message to reach the PT independently of pineal melatonin in mammals. The melatonin-independent photoperiodic entrainment of the annual thyroid-stimulating hormone (TSH) rhythm was reported in the European hamster, suggesting the presence of the non-visual DBPs in mammals (Saenz De Miera et al., 2018). Interestingly, encephalopsin (Opn3) was found to be expressed in different areas of the rodent brain, indicating a potential role of Opn3 in the non-visual photic process due to the changes in light (Blackshaw and Snyder, 1999; Nissila et al., 2012).

The initiation of light-induced physiological change is particularly important for diurnal animals such as mammals and birds. However, those within the avian brain have not been studied extensively. In this study, the author explored and derived how non-visual photoreceptive cells in the avian brain may connect to circuits controlling the aspects of feeding and emotional behaviors, which will provide an intriguing perspective on how environmental light can be a critical cue for the welfare of birds.

## Effect of Light on the Behavior and Physiology of Birds

Light information characterizing the particular day length (i.e., photoperiod) and intensity can be stored within the organism and subsequently used to provide time signals for the adjustments of the physiological behavior of animals (Farner and Wingfield, 1980; Gwinner, 1989; Brandstatter et al., 2000). Animals must be able to discriminate between short and long days to perform photoperiodic time measurement. The differences of circadian changes related to the reproductive activation between mammals and avian species were well-reviewed by recent reports (Ikegami and Yoshimura, 2013; Kuenzel et al., 2015; ViviD and Bentley, 2018). In comparison with mammals, the avian circadian pacemaking system seems to be more complicated, being composed of at least three major components containing autonomous circadian oscillators as follows: the pineal gland, the retina, and a central nervous hypothalamic component possibly equivalent to the mammalian suprachiasmatic nucleus (SCN). The avian pineal organ contains photoreceptors with different photopigments including melanopsin (Opn4, an opsin-based photopigment), and synthesizes and secretes melatonin which is regulated by light (Sato, 2001; Kang et al., 2007, 2010).

The effects of artificial light on wild birds are critical for their various biological responses. Especially, artificial light at night alters natural light/dark cycles to be problematic for many avian species, suggesting that disrupting circadian rhythms causes multiple direct and indirect physiological consequences of birds because the unnatural sleep deprivation is associated with cardiovascular disease and endocrine disruption and has a profound effect on the circadian expression of genes associated with the immune and stress response (Dominoni et al., 2016).

Light intensity has a significant effect on the behavior, diurnal activity, and immune function of chickens (Blatchford et al., 2009). When birds are in the higher light intensity, they show a more dramatic circadian rhythm, spending more time active, eating and drinking, walking, foraging, and preening during the photophase (light), and resting more time during the scotophase (dark) compared with birds kept at lower light intensities (Alvino et al., 2009; Blatchford et al., 2009; Rault et al., 2017). The rhythms of the multiunit neuronal activity in the premammillary nucleus (PMM) of the caudal hypothalamus of temperate zone bird were demonstrated to show the photoperiod-dependent durations of high activity (Kang et al., 2007; El Halawani et al., 2009). Moreover, in the follow-up confirmation study, low light intensity (10 lux) could not activate PMM in the turkey hypothalamus even in long-day photoperiod (Moore et al., 2018), indicating that light intensity is also a key stimulant of initiation of avian reproductive function as well as photoperiod in avian species. Melanopsin (Opn4) is one of the DBPs which was characterized in the PMM of female turkey (Kang et al., 2007, 2009, 2010; El Halawani et al., 2009; Leclerc et al., 2010).

## Avian DBP Opn4 For Light Perception

The primary system to detect avian photoperiodic information has been thought to be non-retinal, non-pineal DBPs (Benoit and Assenmacher, 1953; Menaker et al., 1970; Yokoyama et al., 1978). Three DBPs (i.e., Opn4, Opsin 5, and Vertebrate ancient opsin) were proposed in the avian brain that responds to photoperiodic information affecting the onset and development of the reproductive function, and all three types of DBPs appear to be involved in priming the neuroendocrine system to activate the reproductive functions of birds (Halford et al., 2009; Kang et al., 2010; Nakane et al., 2010; Kang and Kuenzel, 2015). In this study, the author focused only on Opn4.

Melanopsin (Opn4) was first discovered by Provencio et al. (1998) in the photosensitive melanophores of *Xenopus* skin. *In situ* hybridization studies demonstrated that Opn4 mRNA is also expressed in other photosensitive tissues, such as the retina, the magnocellular preoptic nucleus, and the SCN in the brain (Brown and Robinson, 2004). Later, several studies make Opn4 an attractive candidate for circadian photopigment and non-visual photic responses (Gooley et al., 2003; Hannibal et al., 2013). In non-mammalian vertebrates, Opn4 has two isoforms, namely, mammal-like Opn4m and *Xenopus*-like Opn4x (Bellingham et al., 2006).

Avian Opn4 expression and functional role in the photoperiodic activation of reproductive function were reported in several avian species (Bailey and Cassone, 2005; Kang et al., 2010; Potter et al., 2018; Nakane et al., 2019). A recent study on Japanese quail showed the possible functional role of Opn4 in the mediobasal hypothalamus by evaluating an action spectrum for the expression of photoperiodically controlled beta subunit of TSH in the PT of the pituitary gland (Nakane et al., 2019). Interestingly, it has been suggested that Opn4 may have additional physiological roles beyond the reproductive system in the Pekin duck (Van Wyk and Frakey, 2021).

In mammals, specific populations within PMM were genetically defined as dopaminergic (DAergic) neurons and activated in specific social contexts and functions *via* glutamate release to regulate social interactions; moreover, mammalian PMM has a projection of the catecholaminergic input from locus coeruleus (LoC) (Sobrinho and Canteras, 2011; Soden et al., 2016).

**Figure 1:**
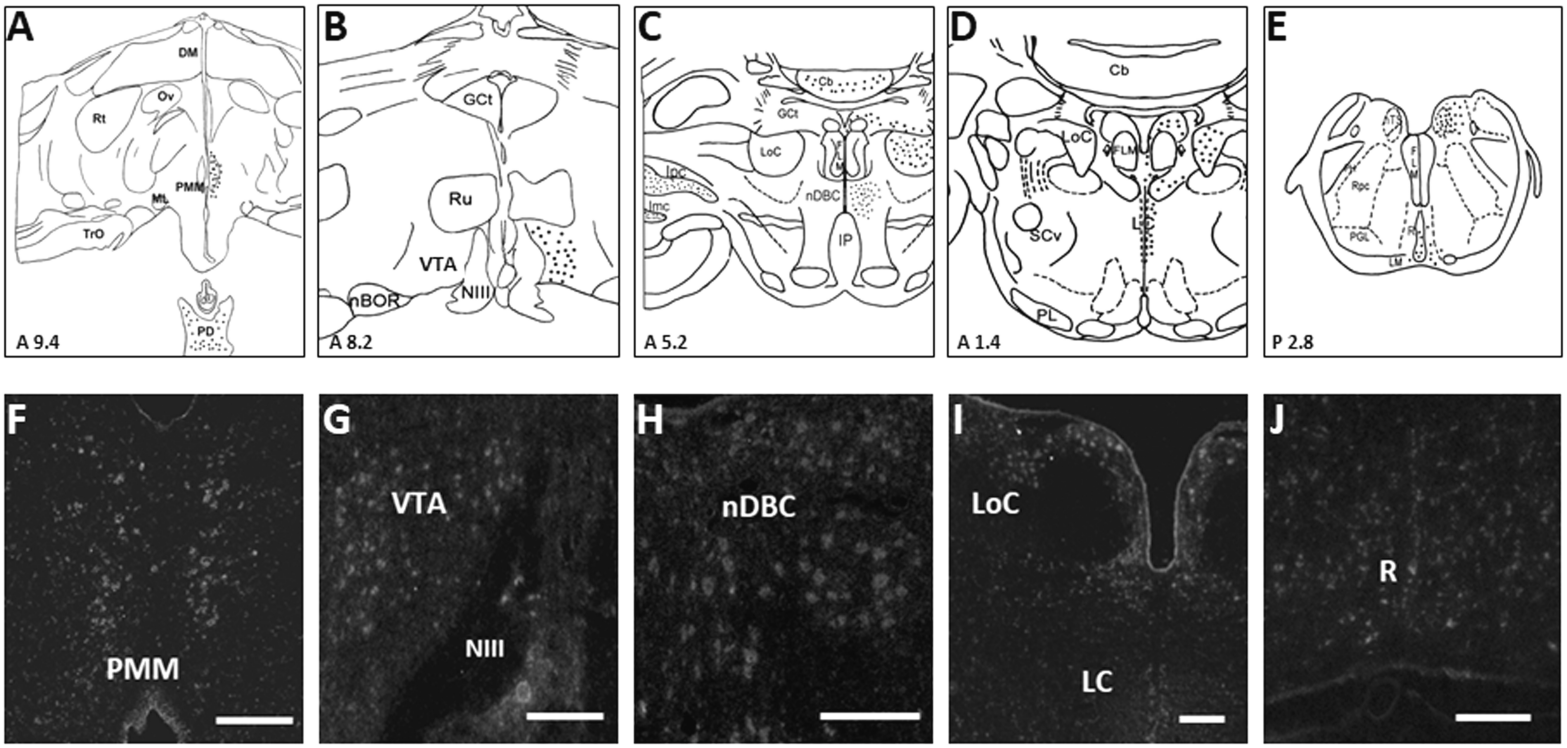
Schematic drawings of rostral-to-caudal **(A–E)** coronal sections, showing the distribution of turkey *Xenopus*-like melanopsin (tOpn4x) expression labeled neurons (filled circles) of the turkey hen. Coronal illustrations were drawn from an unpublished turkey brain atlas with nomenclature taken from a chicken atlas (Kuenzel and Masson, 1988) and the revised nomenclature for avian brains (Reiner et al., 2004). Representative photomicrographs **(F–J)** showing the distribution of tOpn4x mRNA labeled neurons in the PMM, VTA, nDBC, LoC, LC, and R (refer to the abbreviations given below). A specific tOpn4x cRNA probe was used for *in situ* hybridization histochemistry (ISH). Darkfield photomicrographs of turkey brain sections processed for ISH with ^33^P-labeled tOpn4 antisense cRNA probes. Scale bar: 100 μm **(G,H,J)**, 200 μm **(F,I)**. The following abbreviations are used in the figure: Cb, cerebellum; DM, dorsomedial hypothalamic nucleus; MLF, medial longitudinal fasciculus; GCt, mesencephalic central gray; Ipc, parvocellular nucleus Isthmi; Imc, magnocellular nucleus isthmi; IP, interpeduncular nucleus; LC, caudal linear nucleus; LM, medial lemniscus; LoC, locus coeruleus; ML, lateral mammillary nucleus; nBOR, nucleus of the basal optic root; nDBC, nucleus decussationis brachiorum conjunctivorum; NIII, third cranial nerve; nTS, nucleus of the solitary tract; Ov, nucleus ovoidalis; PD, pars distalis; PH, plexus of Horsley; PL, lateral pontine nuclei; PMM, premammillary nucleus; R, raphe nucleus; Rpc, parvocellular reticular nucleus; Rt, nucleus rotundus; Ru, nucleus ruber; SCv, nucleus subcoeruleus ventralis; TrO, tractus opticus; VTA, ventral tegmental area (Modified from Kang et al., 2010).

Avian PMM neurons co-express both dopamine and melatonin (DA-MEL, Kang et al., 2007) and are activated by light provided during the photosensitive phase for reproductive stimulation (Thayananuphat et al., 2007b). The regulation of rhythmic DAergic/melatoninergic (MELergic) activity may involve clock genes, which localize and cycle rhythmically within DA/MEL neurons (Leclerc et al., 2010). Moreover, light pulses that are provided during the photosensitive phase for reproductive stimulation activate these neurons, as indicated by the induction of *c-fos* (Thayananuphat et al., 2007a) and the upregulation of *Cry1* and *Per3* genes (Leclerc et al., 2010). Dopamine and MEL expressing neurons of avian PMM have been shown to have dual functionality, which consists of sensory of light information by Opn4 and neurosecretory functions by the diurnal activities of DA and MEL (Kang et al., 2007, 2009, 2010; Figures 1A,F), suggesting that PMM may be a conserved dual sensory-neurosecretory unit in avian species as suggested in the lower vertebrates (Tessmar-Raible et al., 2007; Conzelmann et al., 2013).

## Opn4 Expression in the Dopaminergic and Serotonergic Nuclei and its Possible Roles in the Welfare of Birds

Photoreceptor Opn4 was observed in the brain areas that are associated with DA and serotonin [5-hydroxytryptamine (5-HT)] in birds (Kang et al., 2010), which were not appreciated hitherto (Figures 1B–E,G–J). It may be of interest to speculate that direct light perception may be involved in the physiological function of DA and 5-HT neurons in the avian brain. Light-induced feed intake in birds may be directly stimulated by central Opn4 because tryptophan hydroxylase 2 (TPH2: rate-limiting enzyme of serotonin biosynthesis) in the dorsal raphe nucleus (DRN) is also associated with food intake and energy balance (Flores et al., 2018; Liu et al., 2021).

Dopamine is predominantly synthesized in the ventral tegmental area (VTA) and substantia nigra (SN) of the midbrain. Dopaminergic neurons in the VTA integrate complex inputs to convert multiple signals that influence motivated behaviors *via* various neural projections underlying the different functions of these neurons in psychological processes and brain diseases (Beier et al., 2015; Bouarab et al., 2019). In mammals, the important roles of DA neurons were discovered in numerous behavioral or psychological processes other than rewards, such as aversion, depression, fear, social behavior, stress, and movement coordination (Pani et al., 2000; Bromberg-Martin et al., 2010; Zweifel et al., 2011; Lammel et al., 2012; Chaudhury et al., 2013; Matsumoto and Takada, 2013; Friedman et al., 2014; Walsh et al., 2014; Grace, 2016; Holly and Miczek, 2016). The major brain structures associated with positive emotion are the amygdala complex and nucleus accumbens (Janak and Tye, 2015). Importantly, the nucleus accumbens is the terminal site of the DAergic mesolimbic axis originating in the VTA (Ikemoto, 2007; Holly and Miczek, 2016). Ventral tegmental area neurons have long been implicated in feeding behaviors, and major neurons are DAergic neurons (about 60% of VTA neurons) (Ungless and Grace, 2012; Meye and Adan, 2014). In addition to DAergic neurons, VTA also contains gamma-aminobutyric acid (GABA) and glutamate neurons that account for about 35 and 2–3% of VTA neurons, respectively (Nair-Roberts et al., 2008; Taylor et al., 2014; Miranda-Barrientos et al., 2021). Besides DA, GABA, and glutamate neurons, several studies reported serotonergic (5-HTergic) neurons in the VTA of mammalian and avian brains (Kang et al., 2009; Carkaci-Salli et al., 2011; Morales and Margolis, 2017; Smith et al., 2019). Interestingly, the optogenetic activation of VTA GABAergic neurons stimulates food intake and anxiety-like behavior in mice (Chen et al., 2020).

The avian VTA contains cell bodies that label positively for tyrosine hydroxylase (TH; the rate-limiting enzyme in catecholamine biosynthesis) but not DA-β-hydroxylase (which is involved in converting DA to norepinephrine), indicating that the major population of avian VTA is DAergic neurons (Kang et al., 2009, Figure 2). The electrophysiological and pharmacological properties of VTA neurons have been studied using whole-cell recordings in the brain slices of birds (zebra finch) (Gale and Perkel, 2006), showing that zebra finch VTA DAergic neurons possess physiological properties very similar to those of mammalian DAergic neurons and also contain non-DAergic neurons similar to GABAergic neurons in the mammalian VTA. In addition, avian VTA DAergic neurons densely innervate the striatal areas of the basal ganglia and project more moderately to several other regions of the telencephalon, and the pharmacological agents and lesions targeting the DAergic system have many similar behavioral effects in birds and mammals (Durstewitz et al., 1999). Therefore, these results provide strong evidence for anatomical, physiological, and functional similarities between the VTA DAergic systems of mammals and birds (Gale and Perkel, 2006).

**Figure 2:**
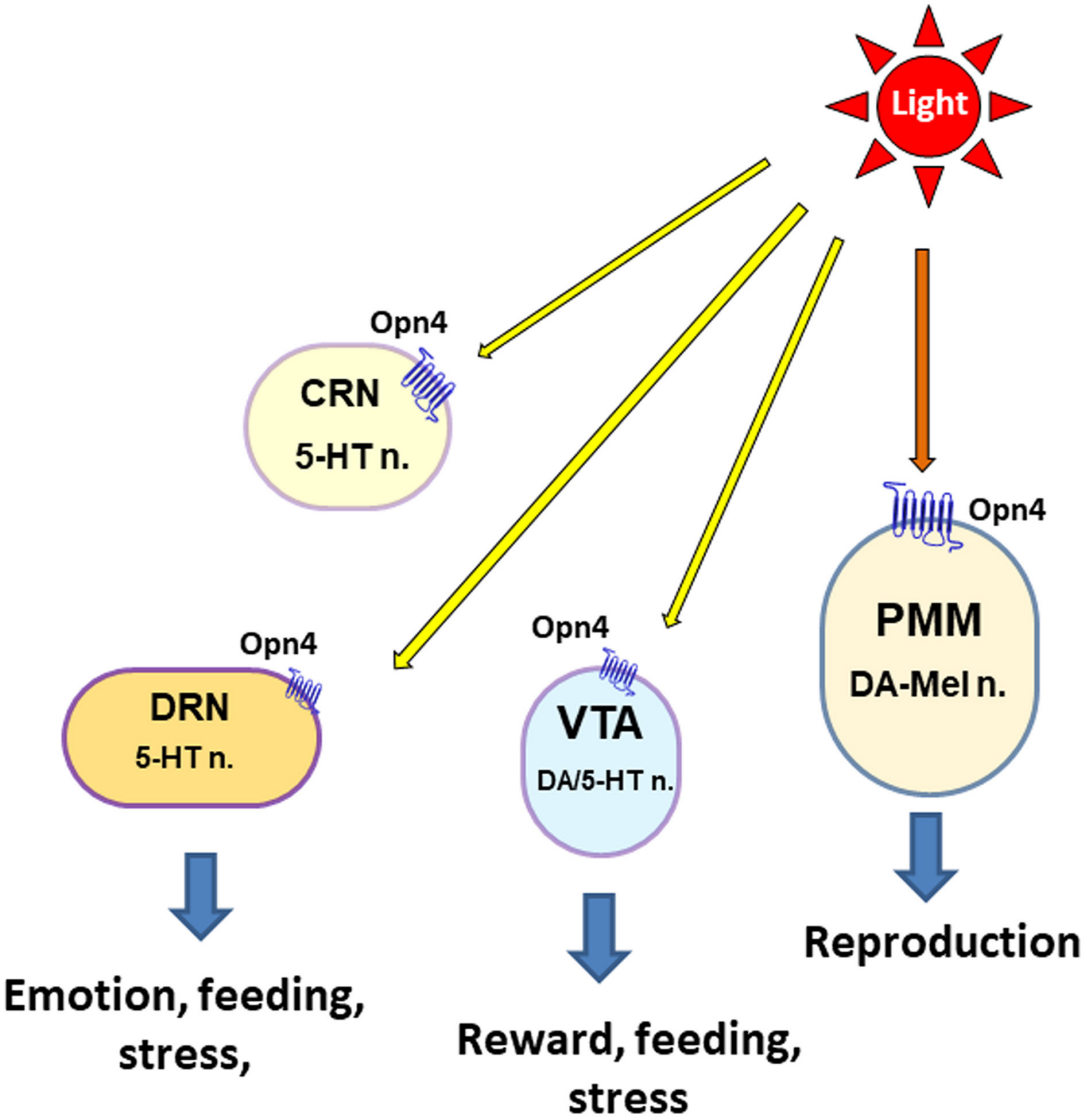
Schematic overview of extraocular light perception in the midbrain and brain stem of avian species for the physiological response. The following abbreviations are used in the figure: 5-HT, serotonin; DA, dopamine; CRN, caudal raphe nucleus; DRN, dorsal raphe nucleus; n, neuron; PMM, premammillary nucleus; VTA, ventral tegmental area.

The distribution of 5-HT immunoreactivity and TPH2 mRNA expression was reported in the avian brain such as VTA, DRN, and caudal raphe nucleus (CRN) (Cozzi et al., 1991; Challet et al., 1996; Kang et al., 2009). The presence of TPH2-positive neurons in the VTA may provide an area of further investigation involving interactions between 5-HTergic and DAergic systems within the VTA (Carkaci-Salli et al., 2011). Serotonin is one of the main neurotransmitters to regulate the parasympathetic nervous system (PNS) and is involved in emotional states caused by stress, pain, or the availability of food (Chamas et al., 1999; Mosienko et al., 2012), while DA acts on the sympathetic nervous system (SNS). Serotonergic neurons can be identified based on the presence of TPH2 mRNA expression, and thereby the TPH2 expression levels can be used as a specific marker for 5-HT generation (Chamas et al., 1999; Kang et al., 2009, 2020; Carkaci-Salli et al., 2011; Liu et al., 2021). The DRN is a heterogeneous brain stem nucleus located in the midbrain and pons, which is involved in the control of various physiological functions, such as learning and memory (Michelsen et al., 2008). The most abundant neurotransmitter in the DRN is serotonin, and the TPH2 mRNA expression was observed in the avian DRN such as nucleus decussationis brachiorum conjunctivorum (nDBC), LoC, and caudal linear nucleus (LC) (Kang et al., 2009, Figure 2).

The presence of both DA and 5-HT systems in the VTA indicates that avian VTA is the critical area of the midbrain involved in the welfare of avian species (Kang et al., 2009, 2020; Carkaci-Salli et al., 2011). Several studies have proposed that DA and 5-HT could serve as positive indicators of animal welfare (Algers et al., 2007; Boissy et al., 2007; Polter and Kauer, 2014). Stress and negative experience alter the 5-HT metabolism in the brain by stimulating 5-HT turnover in the areas innervated by 5-HTergic neurons (Clement et al., 1993; Inoue et al., 1994; Amat et al., 1998). In mammals, repeated immobilization stress increased the *TPH2* gene expression levels in the raphe nuclei of the brain stem (Chamas et al., 1999; Walther et al., 2003), indicating the elevation of 5-HT metabolism. In the recent study of DA and 5-HT activity, 5-HTergic and DAergic activities respond differently to light intensity and light intensity preference, and these results suggest the beneficial effects of dual intensity lighting program on the protection of the central nervous system of birds (Kang et al., 2020).

## Perspective

Animals explore their surroundings to secure resources such as food, water, and shelter, and the regulation of their reproductive system for producing offspring depends on the environment day-and-night light condition.

The data discussed in this study and the previous light intensity study (Kang et al., 2020) suggest the possible roles of Opn4 in the VTA and DRN/CRN on the direct light perception for the physiological responses of birds such as feeding behavior and welfare. Although this observation makes the hypothesis that Opn4 is a positive candidate photoreceptor associated with direct light perception in the ancient brain (i.e., hypothalamus and brain stem) of birds, the functional role of Opn4 should be tested in the future study.

## Data Availability Statement

The original contributions presented in the study are included in the article, further inquiries can be directed to the corresponding author/s.

## Author Contributions

SK contributed to the conception, drafted the manuscript, edited and revised the manuscript, and approved the final version of the manuscript.

## Funding

This study was financially supported by the US Poultry and Egg Association Research Grant.

## Conflict of Interest

The author declares that the research was conducted in the absence of any commercial or financial relationships that could be construed as a potential conflict of interest.

## Publisher's Note

All claims expressed in this article are solely those of the authors and do not necessarily represent those of their affiliated organizations, or those of the publisher, the editors and the reviewers. Any product that may be evaluated in this article, or claim that may be made by its manufacturer, is not guaranteed or endorsed by the publisher.
